# Risk Stratification of Endoscopic Submucosal Dissection in Colon Tumors

**DOI:** 10.3390/jcm11061560

**Published:** 2022-03-12

**Authors:** Katarzyna Winter, Marcin Włodarczyk, Jakub Włodarczyk, Igor Dąbrowski, Ewa Małecka-Wojciesko, Adam Dziki, Michał Spychalski

**Affiliations:** 1 Center of Bowel Treatment, 95-060 Brzeziny, Poland; igordabrowski@onet.eu (I.D.); mspych80@gmail.com (M.S.); 2 Clinical Department of General and Oncological Gastroenterology, University Clinical Hospital No. 1, Medical University of Lodz, 90-153 Lodz, Poland; 3 Department of General and Oncological Surgery, Medical University of Lodz, 90-153 Lodz, Poland; dr.mwlodarczyk@gmail.com (M.W.); dr.jwlodarczyk@gmail.com (J.W.); 4 Department of Digestive Tract Diseases, Medical University of Lodz, 90-153 Lodz, Poland; ewuncia@poczta.onet.pl; 5 Department of General and Colorectal Surgery, Medical University of Lodz, Haller Square 1, 90-419 Lodz, Poland; adam.dziki@umed.lodz.pl

**Keywords:** ESD, colon tumors, complication rate, success rate

## Abstract

Background: Endoscopic submucosal dissection (ESD) is a technique proven effective in the treatment of early neoplastic lesions in the gastrointestinal tract. However, in the case of colon lesions, many doubts remain. The purpose of our study is to stratify the success rates of the ESD procedure in the colon. Materials and Methods: A retrospective analysis of 601 patients who underwent ESD procedure for colorectal neoplasm from 2016 to 2019 in Center of Bowel Treatment, Brzeziny, Poland. Excluding 335 rectal neoplasms, we selected 266 patients with lesions located in the colon. Results: Lesions located in the left colon were characterized by the statistically higher en bloc resection and success rate, compared with the right colon—87.76% vs. 73.95% (*p* = 0.004) and 83.67% vs. 69.75% (*p* = 0.007), respectively. The success rate was significantly lower in lesions with submucosal cancer, compared to low- and high-grade dysplasia (*p* < 0.001). Polyps located in the right colon were characterized by a slightly higher complication rate compared to the left colon, without statistical significance—13.45% vs. 9.52% (*p* = 0.315). Conclusions: Our results show that colonic ESD has a high success rate, especially in the left colon, with a low risk of complications, slightly higher than in the right colon.

## 1. Introduction

Colorectal endoscopic submucosal dissection (ESD) has become the standard treatment for larger neoplastic lesions in Japan [1]. The advantage of ESD is higher en bloc and R0 resection rates (compared to endoscopic mucosal resection; EMR) and lower invasiveness (compared to surgery) [2,3,4,5,6]. However, colorectal lesions are a technical and time-consuming challenge, especially in the right colon and within the flexures. Moreover, due to the thinness of the intestinal wall, the method is associated with a higher complication rate (perforation and postoperative delayed bleeding) than mucosectomy [3,7]. In the case of Western countries, probably due to less experience, there is a great concern about implementing this procedure as a routine in the resection of colorectal neoplastic lesions.

The aim of our study was to stratify the success rates of the ESD procedure in the colon. We have made an attempt to identify colonic lesions that are easy to dissect with low risk of procedure-related complications. We based on the analysis of 266 cases of colon polyps excised using the ESD technique by a single operator, following the appropriate, ESGE-compliant learning program.

## 2. Materials and Methods

### 2.1. Study Population

We conducted a single-center, retrospective, cohort study on 601 patients who underwent the ESD procedure for a colorectal neoplasm from January 2016 to December 2019 at the Center of Bowel Treatment, Brzeziny, Poland. We selected, from this group of patients, patients with lesions localized from the rectal sigmoid flexure to the ileocecal valve, excluding ESD performed in the rectum. Data for the study were collected using a retrospective review of medical records, a database of outpatient clinic cards, endoscopic reports and histopathological results.

The exclusion criteria included: inflammatory bowel disease, gastrointestinal stromal tumors, and familial adenomatous polyposis.

This study protocol was approved by the Committee of Bioethics of Medical University of Lodz, Poland.

### 2.2. ESD Procedure

The indications for ESD strictly followed the Japan Colorectal ESD/EMR Guidelines established by the Japan Gastroenterological Endoscopy Society and included: laterally spreading tumors non-glanular (LST-NG) larger than 20 mm, laterally spreading tumors glanular (LST-G) larger than 30 mm (especially mixed-nodular type), V pit pattern or suspicion of superficial submucosal invasion, and lesions difficult to resect with conventional EMR, i.e., lesions with a non-lifting sign, with submucosal fibrosis or a local recurrence after earlier endoscopic treatments [1].

ESD was performed using the following procedures, as previously described [1,2,8]. Saline or mannitol along with indigo carmine was injected under the submucosa to raise the mucosa layer. An initial circumferential mucosal incision was made around the lesion and then dissection was performed using the Flush Knife-BT (Fujifilm, Tokyo, Japan) and/or Dual Knife (Olympus Medical Systems, Tokyo, Japan). All procedures were performed with a standard Pentax 90i colonoscope with a transparent hood and CO_2_ inflation system. All procedures were performed with deep intravenous sedation or general anesthesia with endotracheal intubation, under the supervision of an anesthesiologist. In most cases, ESD was performed with the VIO300D (ERBE Elektromedizin, Tuebingen, Germany) as a power source for electrical cutting and coagulation. To prevent delayed bleeding, careful additional coagulation and/or hemoclips were used at the end of preparation. Clipping was also used to close any suspected or visible microperforation.

Resected specimens were pinned with needles on a corkboard, and the size of the lesion was measured. Then the specimens were immersed in 10% formalin and sectioned serially in 2 mm intervals for histological evaluation. Vienna classification was used to classify the colon samples. En bloc resection was defined as the excision of the whole lesion in one piece with a healthy tissue margin. Complete histological resection (R0) was defined as surgical excision with healthy margins confirmed by histopathology. Curative ESD procedure or success rate was defined as resections with negative lateral and vertical margins, no poorly differentiated or mucinous histology, no lymphovascular involvement and tumor budding, depth of submucosal invasion <1000 μm below the muscularis mucosae.

After the ESD procedure, patients were kept on a clear liquid diet and fed a light meal during the first post-procedural day. The vast majority of patients were discharged from the hospital after 2–3 days.

### 2.3. ESD Complications

Complications were defined as intraprocedural (detected during the ESD) or delayed (detected post-ESD). Perforation was defined as defects with visible serosa or intraperitoneal tissue, detected during the procedure or as free air in the abdominal cavity on X-ray or CT scan. Perforations detected during the procedure were immediately closed with a metal clip. While delayed perforations were qualified for urgent surgery.

Bleeding during the ESD procedure was not considered a post-procedural complication. Post-ESD bleeding was defined as mild, requiring endoscopy and pharmacological treatment, without blood transfusion, and severe with loss of ≥2 hemoglobin units.

### 2.4. Statistical Analysis

Statistical analysis was performed using Statistica 13.1 (StatSoft, Inc., Tulsa, OK, USA). Numerical data are presented as mean ± SD. The Student’s *t*-test was used for comparison between groups, as well as the nonparametric Mann–Whitney test, depending on the distribution of variables, and the chi-squared test or Fischer test. In all the analyses, differences of *p* < 0.05 were considered statistically significant.

## 3. Results

For the study, we selected 601 patients who underwent colorectal ESD procedures from 2016 to 2019 at the Center of Bowel Treatment (Brzeziny, Poland). We excluded from our analysis 335 patients with rectal neoplasms, ultimately including 266 patients with lesions in the colon. The baseline characteristics of the study group are presented in Table 1.

### 3.1. Efficacy

To analyze the effectiveness of the ESD procedure, we evaluate the en bloc resection and success rate in each part of the colon. Polyps located in the left colon were characterized by the statistically higher en bloc resection and success rate, when compared with the right colon—87.76% vs. 73.95% (*p* = 0.004) and 83.67% vs. 69.75% (*p* = 0.007), respectively. Polyps located in the sigmoid colon, regardless of their size, were characterized by statistically significantly higher en bloc resection and success rate—89.26% and 84.30%, respectively. While polyps located in the cecum and transverse colon were characterized by the lowest en bloc resection and success rate—70% and 63.33%; 68.42% and 63.16%, respectively. Specific outcomes depending on the localization are presented in Table 2.

We also analyzed whether en bloc resection and success rates depend on the pathological stage of lesions. The success rate was significantly lower in lesions with submucosal cancer (SM1, SM2, SM3), compared to low- and high-grade dysplasia (*p* < 0.001) (Table 3). Interestingly, there was no statistical difference in efficacy between lesions with low- and high-grade dysplasia (*p* = 0.081).

Another clinical problem analyzed was in which part of the colon the ESD would be most effective for small lesions lower than 50 mm with low grade dysplasia. We revealed that the highest en bloc resection and success rates were in the sigmoid and ascending colon and the lowest in the cecum and transverse colon, but without statistical significance (*p* = 0.539 and *p* = 0.418, respectively Table 4).

### 3.2. Safety

Analysis of complications revealed ESD procedure related complications in 30 (11.27%) cases. Delayed bleeding was observed in eight patients (3%) and severe in two of them (0.75%). Perforation occurred in 23 patients (8.60%), but only in five cases required surgery (1.87%). Polyps located in the right colon were characterized by a slightly higher complication rate compared to the left colon—13.45% vs. 9.52%, without statistical significance (*p* = 0.315; Table 5). The lowest complication rate was achieved in the sigmoid and ascending colon—8.26% and 7.84%, respectively (Table 6). Higher perforation rate occurred in the right colon compared to the left colon—13/119 (10.92%) vs. 10/147 (6.8%) and only three (2.52%) vs. two (1.36%) required surgery, respectively (Table 5). Perforation was observed more frequently in transverse colon and cecum—15.79% and 13.33% compared to sigmoid (5.79%) and ascending colon (5.88%), but without statistical significance (*p* = 0.257). More detailed information regarding complication rates is presented in Table 6. There was no significant difference in complication rate depending on the histopathological assessment, possibly due to disproportionate groups.

All cases of postoperative bleeding were successfully treated by an endoscopic procedure that entailed metal clipping and/or electrocoagulation, without the need to convert to open surgery. We have not found any deaths related to the ESD procedure.

## 4. Discussion

Despite many obstacles, the ESD technique in the colon is gaining more and more supporters. However, experts’ opinions are divided - some limit the ESD technique to lesions with a significant risk of submucosa invasion, others recommend this method in the treatment of most lesions in the colon [9]. Supporters of mucosectomy (EMR) emphasize its simplicity, safety, low costs and the possibility of effective treatment of most lesions in the colon [10,11,12,13]. While ESD supporters emphasize the advantages of this method, first of all, in the quality of the obtained histological material, a higher percentage of en bloc and R0 resection, and thus a much lower risk of local recurrence [3,14]. 

In a systemic review and meta-analysis of 11 studies and 4678 patients, the en bloc resection rate of colorectal lesions >20 mm was 89.9% for ESD vs. 34.9% for EMR (*p* < 0.001). The R0 resection rate was 79.6% for ESD vs. 36.2% for EMR patients (*p* < 0.001) [3]. The most important aspect was the recurrence risk, reported in 10 studies—0.7% in the ESD group and 12.7% in the EMR one [3]. Another systemic review presented by de Ceglie et al. [4] analyzed 66 studies with a total number of—17950 lesions (EMR: 11873; ESD: 6077). Higher en bloc resection was achieved in the ESD group—90.5% than in the EMR group—62.8% (*p* < 0.0001). Thereby, the recurrence rate was significantly higher in the EMR than ESD group (*p* < 0.0001). 

Due to the fact that the position of endoscopic dissection in rectal lesions is well established, in our study, we selected only patients with colon lesions, excluding rectal ESD. We revealed that lesions located in the left colon were characterized by the statistically higher en bloc resection and success rate, compared with the right colon—87.76% vs. 73.95% (*p* = 0.004) and 83.67% vs. 69.75% (*p* = 0.007), respectively. Lesions located in the sigmoid colon, regardless of their size, were characterized by statistically significantly higher en bloc resection and success rates—89.26% and 84.3%, respectively. In the left colon, we achieved similar results to those in the rectum, thus confirming the great effectiveness of this method in the colon. Similar results were obtained by Rönnow et al. [15]—en bloc and R0 resection rates were 83% and 64% in the distal colon, and 54% and 59% in the proximal colon, respectively.

Another clinical problem we analyzed was in which part of the colon the ESD would be most effective for small lesions <50 mm with low grade dysplasia. In our material, we revealed that the highest en bloc resection and success rate occurred in the sigmoid and ascending colon, and the lowest in the cecum and transverse colon, but without statistical significance, probably due to the small size of the groups (*p* = 0.539 and *p* = 0.418, respectively).

Among the opponents of ESD procedures, one of the basic arguments against this method is the significantly higher risk of perforation [16,17]. In the past, this complication was almost always associated with the necessity for surgical treatment; therefore, it was a significant obstacle in the development of surgical endoscopy [18,19]. However, the risk of perforation during ESD are significantly reduced with experience and in experienced practitioners rates are now below 6% [7,20,21,22,23]. An extensive review of the literature in MEDLINE, EMBASE, Ovid, CINAHL, and Cochrane concerning the clinical efficacy and safety profile of colorectal ESD, including 13,833 tumors in 13,603 patients, showed immediate and delayed perforation rates of 4.2% and 0.22%, respectively [22]. 

In the vast majority of cases, this complication is recognized immediately and successfully managed by closing the defect with endoscopic metal clips and only a small percentage require surgery. The risk of surgical intervention due to perforation in Saito et al.’s [23] study (1111 cases) occurred in 0.45% of cases and in Lee et al.’s [24] study in 0.6% of cases (499 cases). Similar results have been obtained in European studies. Sauer et al. [25] analyzed their initial results of ESD procedures in colorectal lesions (182 cases). The perforation occurred in 9.2% of cases, without the need for surgical intervention. While the Swedish authors Rönno et al. [15], who have analyzed over 300 ESD of colorectal lesions, found a perforation rate of 5.6%. Surgical treatment was required in 2% of patients (all with proximal lesions, due to delayed perforation). In the current study limited to colonic lesions, perforation occurred in 23 patients (8.6%), but only in five cases required surgery (1.87%). Lower perforation rate occurred in the left colon compared to the right colon—10/147 (6.8%) vs. 13/119 (10.92%) and only two (1.36%) vs. three (2.52%) required surgery, respectively. In the right colon, the highest perforation rate was observed in the transverse colon—15.79%. While in the left colon the lowest perforation rate was achieved in the sigmoid colon—5.79%. 

Another common post-ESD complication is bleeding. In our study, post-ESD delayed bleeding occurred in eight patients (3%) and was severe in two of them (0.75%). Delayed bleeding was more often in the left colon—3.4%, especially in sigmoid—3.31% vs. ascending colon—1.96%, but without statistical significance. All cases of postoperative bleeding were successfully treated by an endoscopic procedure, without the need to convert to open surgery. Similar results were obtained by Yamamoto et al. [7] where post-ESD bleeding was reported in 19/398 patients (4.8%). Similarly, to our results, post-ESD bleeding was influenced by neither the invasion depth nor the size of the lesion. In a systematic review and meta-analysis performed by Akintoye et al. [22], which included 13,833 colorectal tumors after ESD, rates of immediate and delayed major bleeding were 0.75% and 2.1%, respectively. Although definitions of significant bleeding vary between studies, it reportedly occurs in 0.5–2.75% of cases [21,22,23].

The growing experience in ESD procedures and the decreasing risk of complication are changing the approach to this procedure. In Japan, patients undergoing ESD are hospitalized for 5–6 days [26]. In uncomplicated cases, the mean hospital stay is decreased to 3.4 days [26]. In European centers, ESD patients are hospitalized for 2–3 days [27]. In our study, the mean hospital stay was 4.56 ± 1.59. However, some of the European endoscopists underline the financial advantage of ESD procedures on an outpatient basis [28]. Probably, some technically simple lesions in near future will be removed in many centers, including ours, in the one-day endoscopy mode. 

While the study presented comprises the largest number of colon ESDs reported from Europe, there are some limitations. The study was conducted in a single-center, by a single-operator, thus, it is difficult to generalize the reported results. In addition, the study has a retrospective design without a control group (e.g., EMR). However, our data show that following the appropriate, ESGE-compliant learning program, it is possible to obtain ESD outcomes close to the experts from the Eastern countries.

## 5. Conclusions

This study suggests that ESD can be used effectively and relatively safely for the treatment of early colon lesions as a common therapeutic approach. Lesions located in the left colon, especially in the sigmoid, are easier to dissect with a slightly higher safety profile. Taking into account these results one needs tailored endoscopic therapy for colonic lesions, where ESD is a valuable option.

## Figures and Tables

**Table 1:** Baseline characteristics of the study group.

Age		64.96 ± 11.16
Sex	Female	45.9% (*n* = 122)
	Male	54.1% (*n* = 144)
Types of polyps	LST-G	51.1% (*n* = 136)
	LST-NG	24.4% (*n* = 65)
	Na	24.4% (*n* = 65)
Paris Classification	IIA	52.3% (*n* = 139)
	IS	24.8% (*n* = 66)
	IA + IS	0.8% (*n* = 2)
	IIA + C	7.5% (*n* = 20)
	IIC	0.4% (*n* = 1)
	IIA + IS	14.3% (*n* = 38)
Localization	Right colon:	44.7% (*n* = 119)
	Cecum	11.3% (*n* = 30)
	Ascending colon	19.0% (*n* = 51)
	Transverse colon	14.3% (*n* = 38)
	Left colon:	55.3% (*n* = 147)
	Descending colon	9.8% (*n* = 26)
	Sigmoid colon	45.5% (*n* = 121)
Histopathology	Minor dysplasia	34.2% (*n* = 91)
	Major dysplasia	46.6% (*n* = 124)
	Intramucosal carcinoma	8.6% (*n* = 23)
	SM1	6% (*n* = 16)
	SM2	3% (*n* = 8)
	SM3	1.5% (*n* = 4)
Tumor size [cm]		4.22 ± 1.53
Mean hospitalization time [days]		4.56 ± 1.59

**Table 2:** Efficacy of ESD procedure in individual parts of the colon.

	Sigmoid Colon	Descending Colon	Transverse Colon	Ascending Colon	Cecum	*p*-Value
En bloc resection	89.26% (*n* = 108)	80.77% (*n* = 21)	68.42% (*n* = 26)	80.39% (*n* = 41)	70% (*n* = 21)	0.018
Success rate	84.30% (*n* = 102)	80.77% (*n* = 21)	63.16% (*n* = 24)	78.43% (*n* = 40)	63.33% (*n* = 19)	0.023

**Table 3:** Efficacy of ESD procedure depending on the histopathology assessment.

	LGD	HGD	Submucosal Cancer	*p*-Value
En bloc resection	75.82% (*n* = 69)	86.39% (*n* = 127)	75% (*n* = 21)	0.079
Success rate	73.63% (*n* = 67)	85.71% (*n* = 126)	46.43% (*n* = 13)	<0.001

LG—low grade dysplasia; HG—high grade dysplasia.

**Table 4:** Efficacy of ESD procedure in lesion <50 mm with LGD according to location of the lesion.

	Sigmoid Colon	Descending Colon	Transverse Colon	Ascending Colon	Cecum	*p*-Value
En bloc resection	85.71% (*n* = 24)	80% (*n* = 8)	66.67% (*n* = 6)	86.36% (*n* = 19)	69.23% (*n* = 9)	0.539
Success rate	85.71% (*n* = 24)	80% (*n* = 8)	66.67% (*n* = 6)	81.82% (*n* = 18)	61.54% (*n* = 8)	0.418

**Table 5:** Complications regarding the location of lesions in the right or left colon.

	Left Colon	Right Colon	*p*-Value
Complication rate	9.52% (*n* = 14)	13.45% (*n* = 16)	0.315
Bleeding	3.4% (*n* = 5)	2.52% (*n* = 3)	0.676
Severe bleeding	6.8% (*n* = 1)	10.92% (*n* = 1)	n/a
Perforation	6.8% (*n* = 10)	10.92% (*n* = 13)	*p* = 0.234
Perforation requiring surgery	2	3	n/a

n/a—not applicable.

**Table 6:** Complications depending on the exact location of the lesions in the colon.

	Sigmoid Colon	Descending Colon	Transverse Colon	Ascending Colon	Cecum	*p*-Value
Complication rate	8.26% (*n* = 10)	15.38% (*n* = 4)	18.42% (*n* = 7)	7.84% (*n* = 4)	16.67% (*n* = 5)	0.293
Bleeding	3.31% (*n* = 4)	3.85% (*n* = 1)	2.63% (*n* = 1)	1.96% (*n* = 1)	3.33% (*n* = 1)	n/a
Severe bleeding	1	0	0	0	1	n/a
Perforation	5.79% (*n* = 7)	11.54% (*n* = 3)	15.79% (*n* = 6)	5.88% (*n* = 3)	13.33% (*n* = 4)	0.257
Perforation requiring surgery	1	1	1	1	1	n/a

n/a—not applicable.

## Data Availability

The data presented in this study are available on request from the corresponding author. The data are not publicly available.
